# Reliability, Learnability and Efficiency of Two Tools for Cement Crowns Retrieval in Dentistry

**DOI:** 10.2174/1874120701812010027

**Published:** 2018-06-29

**Authors:** Cristina Bignardi, Elisabetta M. Zanetti, Mara Terzini, Anna R. Ciccola, Gianmario Schierano, Alberto L. Audenino

**Affiliations:** 1 Department of Mechanical and Aerospace Engineering, Polytechnic University of Turin, Turin, Italy; 2 Department of Engineering, University of Perugia, Perugia, Italy; 3 Department of Surgical Science, Dental School, C.I.R University of Turin, Turin, Italy

**Keywords:** Retrieval, Cement, Compressed air, Inverted hammer, Coronaflex, Crown, Bridge, Removal

## Abstract

**Background:**

Tooth-supported fixed dentures are commonly used in restorative dentistry, and have definitely reached a high survival rate; nevertheless, their removal is sometimes required mainly due to caries or other failures (poor fit, poor cementation and so on). Removing a definitive partial denture is not trivial since the used cement is not always known and it may be very resistant; additionally, there are various clinical circumstances in which a conservative disassembly would be desirable.

**Objective:**

assessing the performance of different tools for cement crowns retrieval in terms of reliability, learnability and efficiency.

**Methods:**

An experimental study has been performed on two different devices for conservative crown/bridge removal: a manual tool that is a sliding hammer, and an automatic tool, powered by compressed air. Both skilled and unexperienced operators have been considered and an experimental set up has been appositely designed in order to measure force versus time patterns. The peak applied force has been taken as an output variable for the evaluation of tool performance.

**Results:**

The automatic tool improves both the inter-operator and the intra-operator reliability, respectively from 79% to 95%, and from 69% to 92%. Additionally, the force pattern is significantly different between these two tools: the instrument powered by compressed air, produces a sharper peak force, as required to break fragile materials such as dental crown cement, and its efficiency can be estimated to be 75% higher. Both tools have a high learnability since the performances of experienced and unexperienced operators have not proved to be significantly different.

**Conclusion:**

A methodology has been set up to compare tools for cement crowns retrieval. The compressed-air tool has been proved to generally provide a better performance unless more ductile cement is to be broken.

## 1 INTRODUCTION

The effectiveness and safety of medical tools depend on various factors such as optimized design [1], proper training, safe use and their suitable application [2]; all of these factors can be optimized through a careful design. These aspects are being emphasized in recent years, both in literature [3, 4] and in directives [5] due to more and more complex instruments being introduced by the medical practice on one side, and to the acquired awareness that a part of medical errors, practitioners stress and patient discomfort and inconveniences could be avoided through a more careful design [6]. A well-conceived and well-designed device should allow health-care practitioners to carry out their work effectively and efficiently and enable patients to have improved health outcomes [4, 7]. These concepts have been applied referring to two different tools for the retrieval of partial dental fixtures, focusing on tool efficiency, reliability, and learnability.

The analyzed tools have been designed to remove a definitive partial denture: tooth-supported fixed dentures are commonly used in restorative dentistry, and have definitely reached a high survival rate (about 90% at 10 years [8]); nevertheless their removal is sometimes required mainly due to caries or other failures (poor fit, poor cementation and so on). Removing a definitive partial denture is not trivial since the used cement is not always known and it may be very resistant; additionally, there are various clinical circumstances in which a conservative disassembly would be desirable [9]. This work has been carried on having considered that the partial denture removal procedure has a significant impact on dentist profession and on patient satisfaction: as a result of repetitive blows to remove the denture, different adverse events can eventually take place: patient discomfort, anxiety or pain [10], dental practitioner stress [10], denture damage or, worst of all, abutment tooth loosening. Therefore, identifying the optimal tool for partial dentures removal is a fundamental step to achieve the maximum patient comfort and satisfaction, with the minimum stress of the dental practitioner [11]. Automatic tools are expected to alleviate some critical issues such as operator bias and variability, however, their efficiency (which is related to the applied force history, mainly) is likely to be different from manual tools and it needs to be evaluated.

A dedicated experimental workbench has been set up and the performances of two common tools have been compared: a sliding hammer, and an air-driven impactor. Other articles have inquired this subject [8, 9, 12]. In particular, the last work reports a comprehensive analysis of existing tools with the respective clinical indication. A complimentary, experimental approach has been followed since the full force versus time pattern has been acquired and reported; the knowledge of the force versus time pattern can be useful to make objective comparisons among different tools, in relation to the mechanical properties of dental cement [13, 14], and to obtain a quantitative index of output variability. This work has involved various dental practitioners with different experiences, having assumed that it cannot be stated *a priori* that benefits/drawbacks coming from using a certain tool that is the same for all operators and comparing operators with different experience could give objective information in relation to tool learnability.

The experimental bench here set up is general and it can be used also for the analysis of other tools for partial denture removal; it allows performing controlled experiments with the maximum repeatability.

## 2 METHODS

The experimentation has involved six operators; three operators have been classified as ‘experienced operators’ as they were dentists with more than five years of experience; the remaining three operators have been classified as ‘unexperienced’ since they were dental students with limited prior experience.

**Fig. (1):**
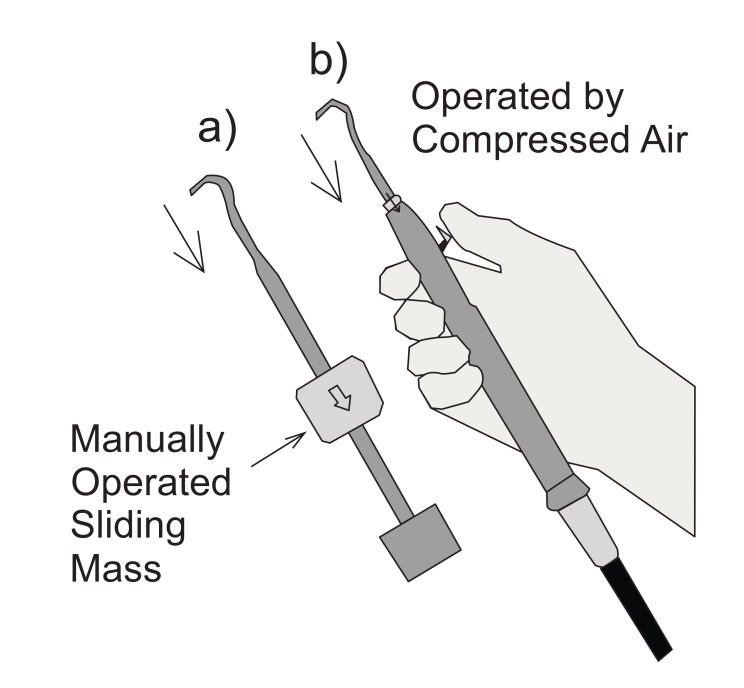
Partial denture removal tools: the manual sliding hammer tool (a) and the automatic air-driven tool (b).

Two different tools have been tested: a manual tool and an automatic tool. The manual tool is a sliding hammer: the impulsive extraction force is created throwing a mass towards the tool handle (Fig. **1**); the applied impulsive force, therefore, depends on the sliding mass speed given by the operator. The automatic tool is an impactor, powered by compressed air; a release button is operated by the thumb in order to produce blows to remove the partial denture.

120 replications of impacts have been produced for each operator/tool combination.

### 2.1 Experimental Bench

**Fig. (2):**
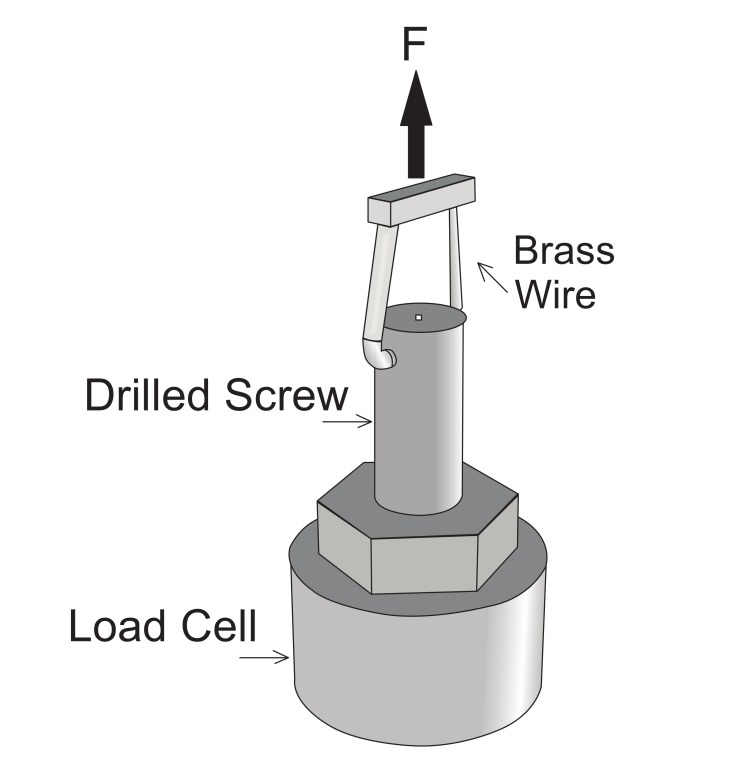
Experimental set-up: the loop passes through the screw hole, and the screw is glued to a load cell.

The most common technique for removing bridges is using a brass wire threaded through bridge embrasures to form a loop onto which a force can be applied to dislodge the bridge. This device is commonly used both with manual and automatic tools. The experimental setup has simulated the partial denture to be removed with a screw bearing a diametral hole (**2**); this screw has been glued to a load cell (Brüel & Kjær, type 8201, Fig. (**2**), lower part). An upward force has been applied on the loop through a loop holder (Fig. **2**, top); this fixture force has been produced alternatively by a manual tool (Fig. **1a**) or by the automatic tool (Fig. **1b**). The activation of both tools results in a sharp movement of the upper end, as indicated by the arrows in Fig. (**1**).

### 2.2 Experimental Design and Statistical Analysis of Data

The experimental plane included two factors: the operator experience *A* (‘Experienced’ or ‘Unexperienced’ operator, *I*=2 levels α_i_, fixed factor) and the tool type *C* (‘manual’ or ‘automatic’, *K*=2 levels γ_k_, fixed factor). The first factor has one more nested factor *B* in order to include differences among operators (*J*=3 levels β_j(i)_, random factor). All operators gave their informed written consensus to participate in this study.

The number of replications (*N*) has been set equal to 120 for all experiments.

Experimental results have been analyzed by ANOVA, after having checked the underlying hypotheses of normality distribution (Lillie test) and equal variances (F test).

Each measurement can be described with a normal distribution, as in the following:

$y_{i,j(i),k,n}=N(\mu +\alpha_{i}+\gamma_{k}+(\alpha \gamma {)}_{i,k};\sigma_{e}^{2}+\sigma_{B(A)}^{2}+\sigma_{B(A).C}^{2})$

Having supposed:

$\sum_{i=1}^{I}\alpha_{i}=0;S_{A}^{2}=\frac{\sum_{i=1}^{I}\alpha_{i}^{2}}{I-1};\beta_{j(i)}=N(0,\sigma_{B(A)}^{2})\forall j(i)\in [1,j];\sum_{k=1}^{K}\gamma_{k}=0;\;S_{c}^{2}=\frac{\sum_{k=1}^{K}\gamma_{k}^{2}}{K-1};$

$\sum_{i=1}^{I}{\left(\alpha \gamma \right)}_{i,k}=0;\forall k\in [1,K]\;\sum_{k=1}^{K}{\left(\alpha \gamma \right)}_{i,k}=0\;\forall i\in [1,I]\;S_{A.c}^{2}=\frac{\sum_{i=1}^{I}\sum_{k=1}^{K}(\alpha \gamma {)}_{i,k}^{2}}{\left(I-1)(K-1\right)}$

$(B\gamma {)}_{j,(i),k\;}=\;N(0,\sigma_{B(A).C}^{2})\forall k\in [1,K]\&\;\forall j(i)\;\in \;[1,J];$

$\varepsilon_{i,j(i),k,n}=N(0,\sigma_{e}^{2})\forall \;i\;\in [1,I],j(i)\;\in \;[1,J],k\in \;[1,K]$

Where:

• *y_i,j(i),k,n_* is the measurement of interest obtained by the j_th_ operator, having i_th_ experience, using the *k_th_* type of tool;

- µ is the overall mean of the measurement;
- α_i_ is the effect of the operator experience on the applied peak force;
- β_j(i)_ is the effect of varying the operator among those belonging to the same group *i*;
- γ_k_ is the effect of the type of tool on the applied peak force;
- αγ_i,k_ is the effect of the interaction between the operator experience *i* and the type of tool *k*;
- βγ_j(i),k_ is the effect of the interaction between the operator *j*, having experience *i,* and the type of tool *k*;
- ɛ_i,j(i),k,n_ is the random error in y_i,j(i),k,n_;
- σ^2^_EFFECT_ is the variance due to a given random EFFECT;

The ANOVA table of expected mean variances has been reported in Table **1**; the sums of squared coefficients of fixed effects can be so calculated:

$S_{A}^{2}=\frac{\sum_{j=1}^{I}\alpha_{i}^{2}}{I-1};\;S_{C}^{2}=\frac{\sum_{k=1}^{K}\gamma_{k}^{2}}{K-1};\;S_{A.C}^{2}=\frac{\sum_{i}^{I}\sum_{k=1}^{K}(\alpha \gamma {)}_{i,k}^{2}}{\left(I-1)(K-1\right)}$

After having estimated mean squares, single variance components and fixed effects coefficients can be calculated through formula reported in Table **2**.

The efficiency of each tool has been related to the peak force because a tool applying a higher force is more likely to remove the partial denture with a lower number of blows. First of all, the influence of the ‘kind of tool’ factor must be tested through ANOVA; if this test produces a positive result, the efficiency can be evaluated as:

$\mathrm{Ef}f_{k}=\mu +\gamma_{k}$

The different efficiency between the air-driven and the sliding hammer tool is equal to:

$\Delta \mathrm{ef}f=\sum_{k=1}^{K}\left|\gamma_{k}\right|$

as an absolute value and it is equal to:

$\Delta \mathrm{eff}\%=\frac{\sum_{k=1}^{K}\left|\gamma_{k}\right|}{\mu }.100$

as a percentage referred to the grand mean µ.

Having assessed the influence of the kind of tool, ANOVA analysis has been repeated, considering each tool singularly, in order to obtain the best estimate of reliability and learnability for each tool since the repeatability error σ^2^_e_ and the impact of operator experience may be different between the sliding hammer and the air-driven tool. These new ANOVA are two-way analyses, having eliminated the ‘tool factor’ (*C*), and its interactions (*AC* and *B(A)·C*).

For a given tool *t*, the respective coefficients α_i,t_ give an index of ‘learnability’ which has been so defined:

$le_{t}\%=(1-\frac{\sum_{i=1}^{I}\left|\alpha_{i,t}\right|}{\mu_{t}}).100$

Where µ_t_ is the average peak force obtained with tool *t* and α_i,t_ are the coefficients of factor A in the respective two-way ANOVA. According to this formula, the learnability reaches 100% when the difference between the performance of experience versus unexperienced operators is null, and it is equal to zero when the average peak force can change up to 100% in relation to different operator experience.

The repeatability or intra-operator reliability coefficient is a measurement of consistency of results obtained by the same operator and the same tool, and it has been so calculated:

$r_{\mathrm{intra},t}=(1-\frac{\sigma e,t}{\mu_{t}}).100$

Where σ^2^_e,t_ is the residual variance in the two-way ANOVA relative to tool *t*, and µ_t_ is the respective average peak force. This coefficient varies between 0 and 100 and a higher value indicates a greater consistency (low variance due to blow repetitions by the same operator, with the same tool), while a null value indicated that the repeatability error can reach 100% of the average peak force.

The inter-operator (*r_inter,k_*) reliability coefficient for a given tool *t* is aimed at defining the degree to which operators are interchangeable:

$r_{\mathrm{inter},t}(1-\frac{\sigma_{B(A).t}^{2}}{\mu_{t}}).100$

This coefficient varies between 0 and 100 and a higher value indicates a greater consistency (null variance due to different operators), while a null value indicates that the variability of results due to different operators performing the same task, can reach 100% of the average peak force.

## 3 RESULTS

Fig. (**3**) reports typical patterns of force versus time. Differences between the automatic and the manual tool are evident: the automatic tool produces a sharper impact with a higher peak force and a steeper force rise. This is not the only difference: experimental tests have soon demonstrated that the automatic tool provides a higher peak force repeatability. These qualitative statements are supported by the more rigorous statistical analysis reported in the following.

### 3.1 ANOVA and Factorial Analysis

Preliminary tests concerning distribution normality have given a positive result for all populations; on the contrary, tests concerning homoscedasticity have given a negative result: the variance of peak force values measured with the manual tool are 3.6 times the one measured with the air-driven tool. ANOVA has been demonstrated to be quite robust to unequal variances if the factorial plane is balanced (same sample size as in the present case) and the sample size is quite large (N=120 in the present case), nevertheless its result should be interpreted with caution (type I error might be underestimated up to 10% [15]), as detailed in the following.

Table **3** reports 3-way ANOVA results: the kind of tool being employed has proved to be highly significant, therefore this result can be considered reliable, even considering data heteroscedasticity. When the automatic tool is employed, a 232 N higher peak force is reached. This implies the air-driven tool is 75% more efficient than the manual tool (the grand mean of the measured peak force is equal to 310 N).

All other parameters (reliability and learnability) have been estimated by two-way ANOVA due to the assessed data heteroscedasticity, as anticipated in the ‘Materials and Methods’ section.

Table **4** reports 2-way ANOVA results for each tool; the manual tool has produced an average peak force equal to 193 N, while the automatic tool has produced an average peak force equal to 427 N. The operator experience has resulted to produce a variation of the peak force equal to ±28.1 N, resulting in a value of learnability *Le_manual_* equal to 71%, but this estimate is assessed with a confidence level *P* equal to 0.11. The operator experience is even less likely to be a significant factor for the air-driven tool (*P*=0.23), and it would result in a learnability *Le_auto_* equal to 96%.

The variance of the peak force among various operators has resulted to be equal to 1496 N^2^ (38.7 N SD) for the manual tool, and to 381 N^2^ (19.5 N SD) for the air-driven tool; the inter-operator reliability is therefore equal to 79% for the sliding hammer and equal to 95% for the air-driven tool.

Also, the experimental error (that is test repeatability) is different between these two kinds of tools, being equal to 3555 N^2^ (59.6 N SD) for the manual tool, and to 926 N^2^ (30.4 N SD) for the air-driven tool. The intra-operator reliability has been calculated for each tool and it has resulted to be equal to 69% for the manual tool and equal to 92% for the automatic tool.

## DISCUSSION AND CONCLUSION

The experimental set up has been designed trying to maximize tests repeatability; other authors [16] performed impacts on cemented crowns since they wanted to compare not only retrievability tools but also different luting cement. This approach has not been followed because replicating cementation could hamper test repeatability, and this could make impossible to detect differences that are smaller than experimental variability; for example, according to [17], the difference between the manual and the compressed-air tool is not significant, while the present work and clinical data [16] are against this finding.

The drawback of a simplified experimental set up compared to clinical data is that measured forces do not replicate the actual force since the damping effect of bone and soft tissue would reduce peak loads. An analysis like the one performed has, therefore, some limits: it cannot allow, for example, assessing the average number of hits required to remove the crown; and the applied peak force has been used as an index of tool efficiency: according to Worni *et al*. the peak force is the key parameter while the average number of hits is not relevant to patient perception of concussion, noise, pain, and unwillingness to use the device [16].

Other articles have inquired this subject [8, 9], however they have followed different approaches and for the first time full force versus time history has been here acquired and reported; the knowledge of the force versus time pattern is fundamental to make objective comparisons among different tools, in relation to mechanical properties of dental cement [13, 14], and to give an index of output variability [18].

The analysis has outlined how the performances of the manual tool and the compressed-air tool are significantly different in terms of efficiency and reliability, while both instruments have a high level of learnability which makes operator experience not relevant or with very small influence. The manual tool produces a less sharp impact, with a lower peak force, and a lower inter-operator and intra-operator repeatability; therefore its efficiency and reliability are lower compared to the air-driven tool.

Obtained results agree with clinical findings reported in [8], where the sliding hammer is considered to be less reliable, and risky for patients with periodontally involved teeth since it could lead to unintended extraction. Damage to porcelain margins is another critical issue related to this tool.

Practical consequences of results reported are that, first of all, the air-driven tool is more likely to be able to break fragile materials; secondly, its performance is more consistent among various dental practitioners, and therefore, more predictable, finally the reproducibility of the performance made by one given operator is higher, and this suggests that its positioning and use is less critical.

## ETHICS APPROVAL AND CONSENT TO PARTICIPATE

Not applicable.

## HUMAN AND ANIMAL RIGHTS

No animals/humans were used for studies that are the basis of this research.

## CONSENT FOR PUBLICATION

Not applicable.

## Figures and Tables

**Fig. (3):**
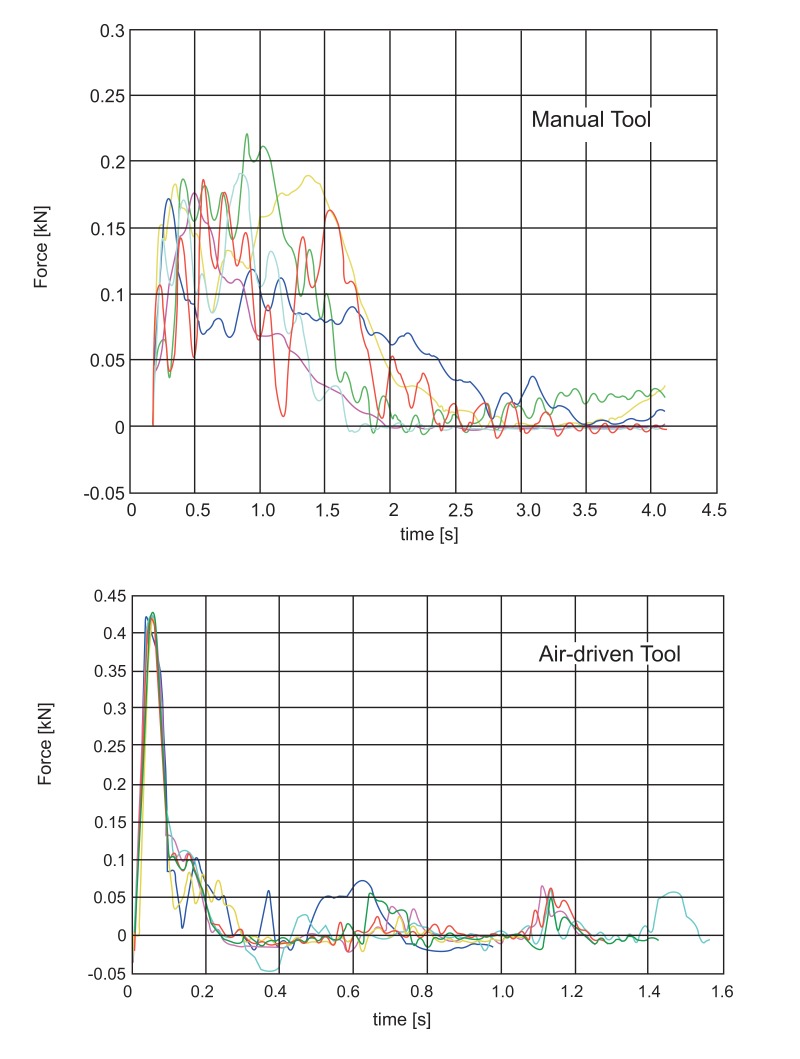
Force versus time curves for the manual tool (up) and for the air-driven tool (down).

**Table 1:** Sources of Variance and their components.

**Source of Variance**	**Degrees of Freedom**	**Observed Mean Squares**	**Expected Mean Squares**	**Denominator** **For F Evaluation**
*A*	(I-1)	MS_A_	+JKN∙S^2^_A_+KN∙σ^2^_B(A)_+ σ ^2^_e_	MS_B(A)_
*B(A)*	I(J-1)	MS_B(A)_	+KN∙ σ ^2^_B(A)_ + σ ^2^_e_	MS_e_
*C*	(K-1)	MS_C_	+IJN∙S^2^_C_+N∙ σ ^2^_B(A)∙C_ + σ ^2^_e_	MS_B(A)∙C_
*A∙C*	(I-1)(K-1)	MS_A∙C_	+JN S^2^_A∙C_+N∙ σ ^2^_B(A)∙C_+ σ ^2^_e_	MS_B(A)∙C_
*B(A)∙C*	I(J-1)(K-1)	MS_B(A)∙C_	+N∙ σ ^2^_B(A)∙C_+ σ ^2^_e_	MS_e_
*Err*	IJK(N-1)	MS_e_	+ σ ^2^_e_	–

**Table 2:** Estimates of Variance Components and of Effects.

**Variances/Effects**	**Estimates**
|α_i_|	[S^2^_A_∙(I-1)/I]^0.5^=[(MS_A_-MS_B(A)_)/JKN∙(I-1)/I]^0.5^
σ^2^_B(A)_	(MS_B(A)_ – MS_e_)/KN
|γ_k_|	[S^2^_C_∙(K-1)/K]^0.5^=[(MS_C_-MS_B(A)C_)/IJN∙(K-1)/K]^0.5^
|αγ_(i,k)_ |	[S^2^_AC_∙(IK-1)/IK]^0.5^=[(MS_AC_-MS_B(A)C_)/JN∙(IK-1)/IK]^0.5^
σ^2^_B(A)∙C_	(MS_B(A)∙C_ – MS_e)_/N
σ^2^_e_	MS_e_

**Table 3:** Results of 3-way ANOVA. Bold characters have been used to point out significant factors (P<0.05).

Source	SS^a^ [N^2^]	DoF^b^	MS^c^ [N^2^]	F^d^	P	Estimated Effect [N] (fixed factors)	Estimated Variance [N^2^] (random factors)
**A (Experience)**	159094	1	159094	5.47	0.08	9.5	
B(A) (Operator)	116381	4	29095	0.15	0.96	/	112
**C (Tool)**	19744040	1	19744040	98.41	**≈0.00**	116.5	/
A∙C	686041	1	686041	3.42	0.14	31.8	/
**B(A)∙C**	802519	4	200630	89.54	**≈0.00**	/	1653
Error	3199574	1428	2241			/	2241
Total	24707648	1439					

^a^ Sum of Squares ^b^ Degrees of Freedom ^c^ Mean Square ^d^ Experimental value of F function

**Table 4:** Results of 2-way ANOVA. Bold characters have been used to point out significant factors (P<0.05).

Manual Tool								
Source		SS^a^ [N^2^]	DoF^b^	MS^c^ [N^2^]	F^d^	P	Estimated Effect [N]	Estimated Variance [N^2^]
A(Experience)		752938	1	752938	4.11	0.11	28.1	1583
**B (Operator)**		732526	4	183132	51.51	**≈0.00**		**1496**
Error		2538599	714	3555				3555
Total		4024063	719	[]				5597
**Air-Driven Tool**								
A(Experience	92197		1	92197	1.98	0.23	8.0	127
**B (Operator)**	186374		4	46593	50.33	≈**0.00**		**381**
Error	660975		714	926				926
Total	939545		719	[]				1307

^a^ Sum of Squares ^b^ Degrees of Freedom ^c^ Mean Square ^d^ Experimental value of F function
